# Wideband Magnetic Excitation System for Atomic Force Microscopy Cantilevers with Megahertz-Order Resonance Frequency

**DOI:** 10.1038/s41598-020-65980-4

**Published:** 2020-06-04

**Authors:** Kaito Hirata, Takumi Igarashi, Keita Suzuki, Keisuke Miyazawa, Takeshi Fukuma

**Affiliations:** 1 Division of Electrical Engineering and Computer Science, Kanazawa University, Kakuma-machi, Kanazawa, 920-1192 Japan; 2 Nano Life Science Institute (WPI-NanoLSI), Kanazawa University, Kakuma-machi, Kanazawa, 920-1192 Japan

**Keywords:** Scanning probe microscopy, Imaging techniques, Characterization and analytical techniques

## Abstract

Small cantilevers with a megahertz-order resonance frequency provide excellent sensitivity and speed in liquid-environment atomic force microscopy (AFM). However, stable and accurate oscillation control of a small cantilever requires the photothermal excitation, which has hindered their applications to the studies on photo-sensitive materials. Here, we develop a magnetic excitation system with a bandwidth wider than 4 MHz, enabling a light-free excitation of small cantilevers. In the system, a cantilever with a magnetic bead is driven by a magnetic field generated by a coil. In the coil driver, a differentiation circuit is used for compensating the frequency dependence of the coil impedance and keeping the current constant. By implementing several differentiation circuits with different frequency ranges, we enable to drive various cantilevers having different resonance frequencies with sufficient excitation efficiency. In contrast to the conventional coil driver with a closed-loop circuit, the developed one consists of an open-loop circuit and hence can be stably operated regardless of the coil design. With the developed system, atomic-resolution imaging of mica in liquid using a small cantilever with a megahertz-order resonance frequency is demonstrated. This development should lead to the future applications of AFM with small cantilevers to the studies on various photo-sensitive materials and phenomena.

## Introduction

Atomic force microscopy (AFM)^1^ has been used for nanoscale imaging of various materials, including metals, semiconductors, photo-catalysts and biological systems^2–6^. This is partly because it can be operated in various environments such as in air, vacuum and liquids. In particular, the capability of in-liquid imaging is useful for the applications in biology and electrochemistry. Among the various operation modes of AFM, there have been significant developments in dynamic-mode AFM for such liquid-environment applications. For example, true atomic-resolution imaging by dynamic-mode AFM was first demonstrated in 2005 by frequency modulation AFM (FM-AFM)^7^. In addition, by combining FM-AFM with three-dimensional (3D) tip scanning methods, subnanometer-scale 3D imaging of hydration structures^8^ and distributions of flexible molecular chains^9^ were demonstrated. Now, such atomic-scale 2D and 3D imaging in liquid have also been demonstrated by other dynamic AFM techniques such as amplitude modulation AFM^10,11^ and bimodal AFM^12–14^. More recently, owing to the developments of commercially-available ultra-small cantilevers with a megahertz-order resonance frequency (*f*_0_), the speed and force sensitivity have been greatly improved^15,16^. Now the applications of these advanced in-liquid AFM technologies are actively explored in a wide range of interfacial sciences.

In dynamic-mode AFM, a micro-fabricated cantilever is mechanically oscillated near its *f*_0_, and the variation in the oscillation amplitude (*A*) or *f*_0_ is detected and used for the tip-sample distance regulation. In this AFM operation mode, the method for exciting the cantilever oscillation is critically important for its accurate and stable operation. To date, various cantilever excitation methods have been developed. Among them, the acoustic excitation^17^ is the most widely used. In this method, a cantilever oscillation is excited by an acoustic wave generated by a piezo-actuator. However, the acoustic wave can excite any spurious resonances existing in the propagation path from the actuator to the cantilever, which can heavily distort the amplitude versus frequency curves^18,19^. This problem becomes particularly serious when AFM is operated in liquid or with a small cantilever^15^.

To solve this problem, various direct excitation methods have been developed, where an excitation force is directly applied to a cantilever without transmission through other mechanical components. Among them, the PT^20^ and magnetic^21,22^ excitation methods have been widely used compared to the other methods. This is mainly because these methods can be used for driving most of the commercially available cantilevers with relatively small modifications even in liquids. For example, the piezoelectric^23–26^, electrothermal^27–31^, and Lorentz force^32^ excitation methods require specially designed microfabricated cantilevers. Compared to them, the electrostrictive^33^ and electrostatic^34–37^ excitation methods have a wider applicability as they only require metal coatings on a cantilever. However, an application of a bias voltage in liquid can induce serious problems such as uncontrolled electrochemical reactions and diffusion of surface charges. These issues hinder its applications to a wide range of liquid-environment studies especially on electrochemical reactions.

In the PT excitation, an excitation laser beam with the power modulated at *f*_0_ is irradiated to the fixed end of a cantilever typically with a metal backside coating. The thermal expansion of the photo-irradiated spot induces mechanical stress in the cantilever and excites the cantilever oscillation. In contrast to the acoustic excitation, a cantilever is directly driven by the laser beam. Thus, we can obtain ideal amplitude versus frequency curves without any influence from the spurious resonances. In addition, owing to the wide bandwidth of the laser power modulation, the method can be easily applied to the ultra-small cantilevers with a megahertz-order *f*_0_^15,16^. Due to these advantages, the use of this method is now rapidly expanding especially in the liquid-environment applications. However, the use of the excitation laser beam can cause serious problems in the investigations of photo-sensitive materials or devices such as photo-catalysts, liquid crystals, cells and light-driven ion channels. Furthermore, the excitation efficiency of this method is relatively low, which often hinders its applications to observe surfaces with relatively large roughness or adhesion.

To avoid this problem, the magnetic excitation method has been used. In this method, magnetic sensitivity is given to a cantilever by coating it with a magnetic thin film^38^ or attaching a magnetic bead^21,22^ on it. By applying an ac magnetic field with a coil integrated in the cantilever- or sample-holder. So far, this method has been widely used for various biological and electrochemical applications^38,39^. However, its applications to an ultra-small cantilever have been impeded by the following technical difficulties.

In the magnetic excitation method, an ac magnetic field is generated by flowing an ac current with a constant amplitude through the coil. This is typically realized by a coil driver with a voltage-to-current (*VI*) converter, where the coil current is continuously adjusted by a feedback controller in proportion to the input voltage signal. The use of an ultra-small cantilever with a high *f*_0_ imposes severe requirements in the stability, bandwidth and output voltage range on the coil driver.

For the stability of the feedback loop, the coil and other electronic circuit components constituting the loop should satisfy requirements on the latency and bandwidth. This becomes more and more difficult as the excitation frequency increases. In particular, these requirements are highly dependent on the characteristics of the coil. Thus, it is more difficult to design a coil driver that is stable for a wide range of coil designs. Another difficulty lies in the excitation efficiency. The impedance of the coil linearly increases with increasing frequency. In addition, as the cantilever size decreases, the area of the magnetic thin film or a size of a magnetic bead decreases, resulting in a low magnetic sensitivity. Therefore, a high-voltage amplifier should be used for the output stage of the coil driver, making it more difficult to satisfy the requirements in the latency, bandwidth and the stability.

In most of the previous applications of the magnetic excitation method, cantilevers with a *f*_0_ less than 300 kHz were used^19,21,22,38–50^. The only exception was reported by Kageshima *et al*.^51,52^, where they developed a wideband coil driver by combining a composite amplifier and a commercially available high-voltage amplifier. They demonstrated the excitation of the third flexural mode of the cantilever at ~700 kHz. However, the magnetic excitation of a cantilever with a megahertz-order *f*_0_ has not been reported.

In this study, we have developed a wideband magnetic excitation system that can drive a cantilever with a megahertz-order *f*_0_ in liquid. In contrast to the conventional coil driver using the feedback loop, here we propose to use an open-loop design with a differentiation circuit for a stable operation even with various cantilevers and coils. In addition, we demonstrate the applicability of the developed system to the atomic-resolution imaging in liquid with an ultra-small cantilever.

## Results and Discussions

### Principle and Basic Setup for Magnetic Excitation

**Figure 1:**
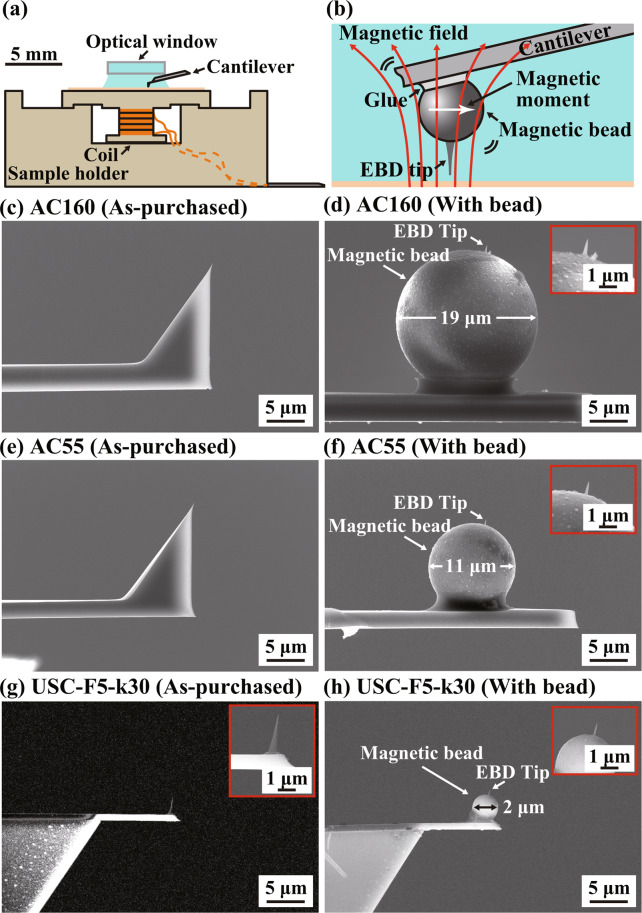
Experimental setup used for the developed magnetic excitation system. (**a**) Cross-sectional view of the sample holder. (**b**) Magnified view of the cantilever free end with a magnetic bead and an EBD tip. (**c–h**) SEM images of the cantilevers used in this study: AC160, AC55 and USC-F5-k30. (**c,e,g**) As-purchased cantilevers. (**d,f,h**) After the bead attachment and EBD tip fabrication.

Figure 1(a) shows a cross-sectional view of the sample holder developed for the magnetic excitation. In this setup, a coil is integrated in a sample holder made of PEEK and placed just under the sample. The coil is fabricated by winding a polyurethane-coated copper wire with a diameter of 0.2 mm by 50 turns around a PEEK cylinder with a diameter of 3 mm and a length of 2.7 mm. The electrical characteristics of the coil and its dependence on the number of turns are shown in Fig. S1 in Supplementary Information.

Figure 1(b) shows a magnified view around the tip. In this study, we used three types of cantilevers: AC160 (Olympus), AC55 (Olympus) and USC-F5-k30 (Nanoworld). Figure 1(c,e,g) show the SEM images of the tips that come with these cantilevers as purchased. AC160 and AC55 cantilevers come with a Si tip while USC cantilever comes with an electron beam deposited (EBD) carbon tip. We removed these tips by focused ion beam (FIB) system (Helios CX, Thermo Fisher Scientific). After the removal, a neodymium bead (MQP-S-11-9-20001-070, Neo Magnequench) was attached to the cantilever end by epoxy glue. The diameter of the bead was approximately 20 *μ* m, 10 *μ*m and 2 *μ*m for AC160, AC55 and USC, respectively. On the bead, an EBD carbon tip with a length of 0.5–1 *μ*m was fabricated by a field emission scanning electron microscope (FE-SEM) (Helios CX, Thermo Fisher Scientific). Figure 1(d,f,h) show the SEM images of the cantilevers after the EBD tip fabrication.

Just before the AFM experiments, the surfaces of the tip, cantilever and magnetic bead were coated with a Si thin film by sputter coater (K’s Tech). The film thickness was 30 nm for AC160 and AC55, and 15 nm for USC. This coating is effective for cleaning the tip surface and provides a stable hydration structure when it is immersed in an aqueous environment^53^. In addition, this coating prevents contamination of the solution by the surface dissolution of the magnetic bead or the EBD tip. Immediately after this coating, a strong magnetic-field pulse generated by a magnetizer (IM10–30, ASC Scientific) was applied to the cantilever in the direction perpendicular to the tip as indicated by the arrow in Fig. 1(b). During the AFM measurements, an ac magnetic field is applied to the tip end with the direction perpendicular to the magnetic moment of the bead, which generates a vertical force to excite the cantilever vibration.

### Open-Loop Coil Driver with Differentiation Circuit

**Figure 2:**
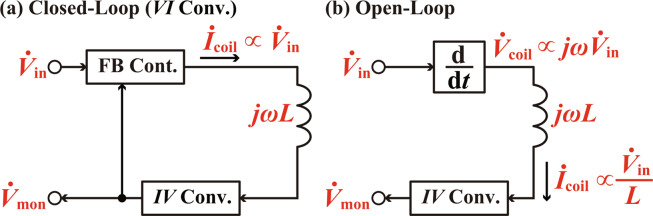
Fundamental designs of the coil drivers. (**a**) Conventional closed-loop design with a *VI* converter. (**b**) Proposed open-loop design with a differentiation circuit.

Figure 2 shows conventional and proposed designs for the coil driver. These coil drivers are used for driving a coil with a constant amplitude ac current (***İ***_coil_) for generating a constant amplitude ac magnetic field without dependence on the driving frequency (*ω*). To achieve this goal, the amplitude of the ac voltage signal applied to the coil should linearly increases with *ω* to compensate the linear increase of the coil impedance *jωL*.

In the conventional design (Fig. 2(a)), ***İ***_coil_ is fed into the current-to-voltage (*IV*) converter to produce the current monitor signal ${\dot{{\boldsymbol{V}}}}_{{\rm{m}}{\rm{o}}{\rm{n}}}$. This signal is routed to the feedback (FB) controller and compared with the input voltage signal ${\dot{{\boldsymbol{V}}}}_{{\rm{i}}{\rm{n}}}$. The FB controller adjusts its output voltage to minimize the difference between the two input signals ${\dot{{\boldsymbol{V}}}}_{{\rm{i}}{\rm{n}}}$ and ${\dot{{\boldsymbol{V}}}}_{{\rm{m}}{\rm{o}}{\rm{n}}}$. In a steady state of this FB control, ***İ***_coil_ is controlled to be proportional to ${\dot{{\boldsymbol{V}}}}_{{\rm{i}}{\rm{n}}}$ regardless of *ω*. This conventional design works fine with a standard cantilever having a relatively low *f*_0_. However, as discussed in Introduction, it is very difficult to achieve a megahertz-order bandwidth without losing stability in the FB control.

To solve this problem, here we propose the open-loop design as shown in Fig. 2(b). In this design, ${\dot{{\boldsymbol{V}}}}_{{\rm{i}}{\rm{n}}}$ is fed into a differentiation circuit to obtain a voltage signal ${\dot{{\boldsymbol{V}}}}_{{\rm{c}}{\rm{o}}{\rm{i}}{\rm{l}}}$ proportional to *jω*${\dot{{\boldsymbol{V}}}}_{{\rm{i}}{\rm{n}}}$. This output voltage linearly increases with *ω* so that it can compensate the frequency dependence of the coil impedance. Thus, the amplitude of ${\dot{{\boldsymbol{I}}}}_{{\rm{c}}{\rm{o}}{\rm{i}}{\rm{l}}}\propto {\dot{{\boldsymbol{V}}}}_{{\rm{i}}{\rm{n}}}/L$ is kept constant irrespective of *ω*.

**Figure 3:**
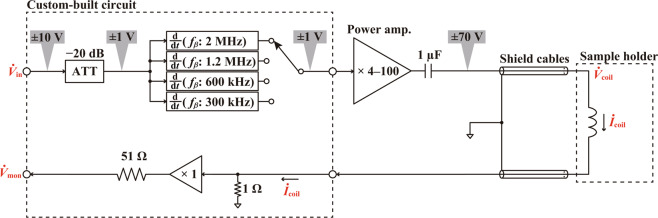
Block diagram of the developed open-loop coil driver with a differentiation circuit.

Figure 3 shows the block diagram of the developed coil driver. In this circuit, the input ${\dot{{\boldsymbol{V}}}}_{{\rm{i}}{\rm{n}}}$ is fed into the −20 dB attenuator to reduce the dynamic range from [−10 V, +10 V] to [−1 V, +1 V]. The attenuated signal is routed to the differentiation circuits with various frequency ranges. One of the outputs of these circuits is selected depending on *f*_0_ to optimize the excitation efficiency and current versus frequency characteristics for each cantilever. Detailed designs for each differentiation circuit are described later. The output of the differentiation circuit is fed into the commercially available high-voltage amplifier (HSA4101, NF). The gain of this amplifier can be adjusted within the range from 4 to 100 while the output range is limited to [−70 V, +70 V]. ***İ***_coil_ is converted to ${\dot{{\boldsymbol{V}}}}_{{\rm{m}}{\rm{o}}{\rm{n}}}$ through 1 Ω resistor for the current monitoring.

In the practical implementation, it is critically important to insert a capacitor at the output of the high-voltage amplifier to eliminate a possible dc offset in ${\dot{{\boldsymbol{V}}}}_{{\rm{c}}{\rm{o}}{\rm{i}}{\rm{l}}}$. Otherwise, even a small dc offset of the amplifier can generate significant heat at the coil. In addition, it is also important to use separate shielded cables for the coil drive and return signal lines as illustrated in Fig. 3. This is for preventing the capacitive coupling between the two lines at the frequency range over 1 MHz.

**Figure 4:**
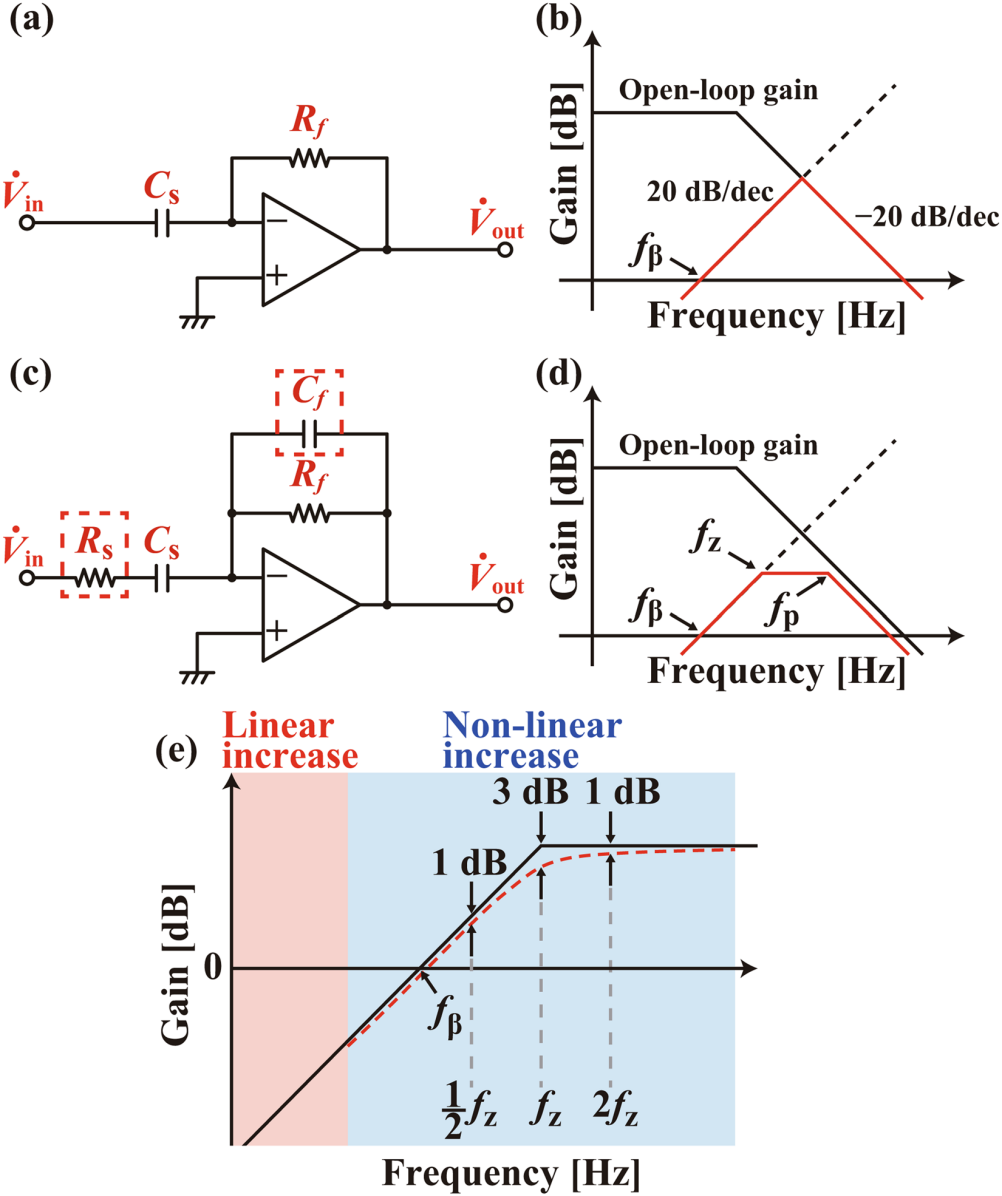
(**a**) Circuit diagram for the basic differentiation circuit. (**b**) The gain characteristics of the Op-Amp and the differentiation circuit shown in (**a**). (**c**) Circuit diagram for the practical differentiation circuit. (**d**) The gain characteristics of the Op-Amp and the differentiation circuit shown in (**c**). (**e**) Magnified view of (**d**) around *f*_*β*_. Solid line shows simplified linear model while the dotted line shows a more realistic model with a non-linear increase of the gain.

Figure 4(a,b) show the circuit diagram of the basic differentiation circuit and its gain versus frequency characteristics, respectively. In this circuit, the output voltage ${\dot{{\boldsymbol{V}}}}_{{\rm{o}}{\rm{u}}{\rm{t}}}$ is given by

1 $${\dot{{\boldsymbol{V}}}}_{{\rm{o}}{\rm{u}}{\rm{t}}}=-j\omega {C}_{{\rm{s}}}{R}_{f}{\dot{{\boldsymbol{V}}}}_{{\rm{i}}{\rm{n}}}.$$

Thus, ${\dot{{\boldsymbol{V}}}}_{{\rm{o}}{\rm{u}}{\rm{t}}}$ increases in proportion to *ω* until it is limited by the open-loop gain of the operational amplifier (Op-Amp) as shown in Fig. 4(b). This circuit shows a gain of 1 at *f*_*β*_(=1/(2*πC*_s_*R*_*f*_)). At this frequency, a pole is formed in the feedback circuit from the output to the inverting input of the Op-Amp. Thus, this circuit starts to oscillate at the frequency where the phase delay within the Op-Amp exceeds 90 deg.

To solve this problem, *R*_*s*_ and *C*_*f*_ are used in a practical differentiation circuit (Fig. 4(c)). *R*_*s*_ and *C*_*s*_ form a zero at *f*_*z*_ (= 1/(2*πC*_s_*R*_s_)), which compensates the phase delay caused by the pole at *f*_*β*_. Above this frequency, the gain curve shows a flat response to the frequency (Fig. 4(d)). In the meanwhile, *R*_*f*_ and *C*_*f*_ form another zero at *f*_p_ = 1/(2*πC*_f_*R*_f_), which not only compensates the phase delay of the Op-Amp but also prevents the gain from being limited by the open-loop characteristics of the Op-Amp. Above this frequency, the circuit works as an integration circuit with a gain slope of −20 dB/dec (Fig. 4(d)). In this way, the addition of *R*_*s*_ and *C*_*f*_ can prevent the oscillation and enables stable operation of the circuit.

The gain of the circuit shown in Fig. 4(c) linearly increases with *ω* at the frequency range much lower than *f*_*z*_, while it shows non-linear response around *f*_*z*_ as shown in Fig. 4(e). By setting *f*_*z*_ at a value much higher than *f*_*β*_ and using the frequency range less than *f*_*β*_ for the coil drive, we can avoid influence from this non-linearity. However, even with such a design, the coil current showed slight increase with *ω*. This is because the impedance of an actual coil does not linearly increase with increasing frequency at a relatively high frequency range. (See impedance characteristics of the coil used for the present work in Fig. S1 in Supplementary Information.) To compensate this non-linear behavior of the coil impedance, we deliberately set *f*_*z*_ relatively close to *f*_*β*_ so that the gain non-linearity can slightly influence the characteristics below *f*_*β*_ as shown in Fig. 4(e). In this way, we optimized the design parameters as shown in Table 1 to achieve the best flatness of the current versus frequency curves for each frequency range.

**Table 1:** Design parameters for the developed differentiation circuits with various frequency ranges.

Freq. Range [MHz]	*R*_s_[Ω]	*C*_s_[*pF*]	*R*_*f*_[*k*Ω]	*C*_*f*_[*pF*]	*f*_*β*_[*MHz*]	*f*_*z*_[*MHz*]	*f*_p_[*MHz*]
(1) 0.06–0.3	680	470	1	22	0.34	0.50	7.23
(2) 0.2–0.8	390	253	1	8	0.63	1.61	19.9
(3) 0.7–2.2	390	133	1	8	1.20	3.07	19.9
(4) 1.4–4.0	510	82	1	4	1.94	3.81	39.8

*f*_0_ of AFM cantilevers have a wide variation. For liquid-environment FM-AFM experiments, *f*_0_ ranges from 0.1 to 4 MHz. If the whole frequency range should be covered by one differentiation circuit, we should use the design parameters adjusted to achieve the highest frequency range. This leads to two major problems. The first issue is on the flatness of the current versus frequency curve. If we adjust the design parameters to achieve the optimal flatness over the whole frequency range, the obtained flatness becomes much worse than that achieved by optimizing the parameters for a local frequency range. The second issue is on the excitation power. As the maximum amplitude of the excitation voltage changes in proportion to 1/*ω*, only a very small excitation amplitude is available at a low frequency range. For example, if the maximum frequency range is set at 4 MHz, the maximum excitation amplitude at 100 kHz is limited to only 1/40 of that at 4 MHz. To solve these problems, we divided the whole frequency range into four ranges (1)–(4) and optimized the design parameters for each of them. By selecting the most appropriate frequency range for each cantilever, we enabled to achieve sufficient performance in the excitation power as well as in the flatness of the current versus frequency characteristics. More specifically, for the cantilevers used in this study, we used the frequency ranges (1) for AC160 (*f*_0_ = 90–110 kHz) and (3) for AC55 (*f*_0_ = 0.9–1.1 MHz) and USC (*f*_0_ = 1.8–2.2 MHz).

### Performance

**Figure 5:**
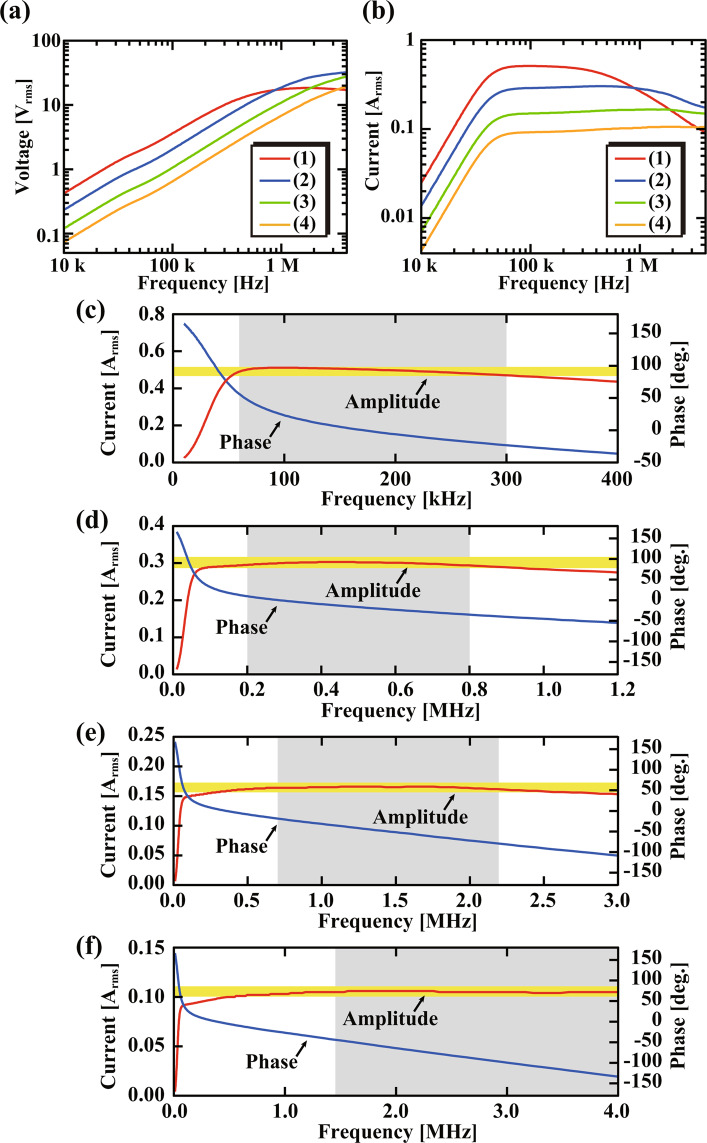
Frequency dependence of the output voltage and current of the developed coil driver. (**a**) Amplitude of the output voltage for the different frequency ranges. (**b**) Amplitude of the output current for the different frequency ranges. (**c–f**) Amplitude and phase of the output current for the individual frequency ranges (1–4). The amplitude range indicated by the yellow background corresponds to the ±5% of the mid-value within each frequency range indicated by the gray background.

Figure 5 shows the frequency dependence of the output voltage and current of the developed coil driver. The measurement was performed with the input voltage amplitude of 10 V, the power amp. gain of 20, and the aforementioned coil (*L* = 5–10 *μ* H) connected to the output. Owing to the frequency characteristics of the differentiation circuit, the output voltage almost linearly increases with frequency at the low frequency range as shown in Fig. 5(a). For all the frequency ranges (1–4), the amplitude of the output voltage at *f*_*β*_ is 10–13 V_rms_ (= 14–18 V_0−*p*_). This is slightly smaller than the expected amplitude (20 V_0−*p*_) due to the influence from the non-linear part in the gain versus frequency characteristics (i.e., influence of the zero at *f*_*z*_). The output gain can be increased up to 100 without causing any problems for the coil driver itself. However, we found that an application of such a large voltage results in a significant increase of the sample temperature. Thus, we decided to use the gain of 20. The temperature issues will be further discussed later with experimental results.

Figure 5(b) shows the output current curves measured with different frequency ranges. This figure shows that the lower current is obtained with a higher frequency range. This is because we used the same power amp. gain of 20 for all the cases in spite of the linear increase of the coil impedance with increasing frequency. Figure 5(c–f) show the magnified views of the amplitude and phase curves for each frequency range. As indicated by the yellow background, the amplitude is kept almost constant with a deviation less than ±5% of the mid-value within each frequency range (gray back ground). In addition, in an actual FM-AFM experiment, the frequency shift caused by the tip-sample interaction is typically less than 1% of *f*_0_. Thus, the current change within such a small range is negligible for most of the AFM applications. The phase curves show almost linear frequency dependence within the specified frequency range, which ensures that the distortion of the output wave form is negligible. These four frequency ranges cover the whole frequency range of 0.1–4 MHz with some overlaps. This allows us to select an appropriate frequency range for various cantilevers with different *f*_0_.

**Figure 6:**
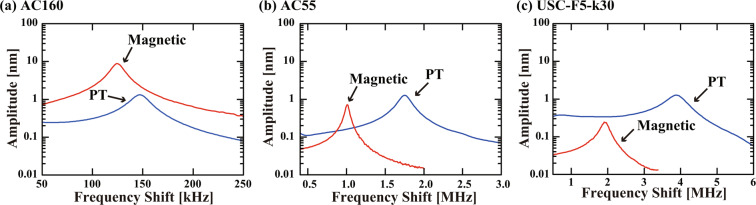
Cantilever oscillation amplitude curves measured in water with different cantilevers including (**a**) AC160, (**b**) AC55, and (**c**) USC-F5-k30. The measurements with the PT and magnetic excitation methods were performed before and after the attachment of a magnetic bead to the cantilevers. For the PT excitation, a 785 nm laser beam was used with a power modulation amplitude of 9.3 mW_p−p_. For the magnetic excitation, the input voltage amplitude of 10 V (i.e., maximum input) and the power amp. gain of 20 were used.

Figure 6 shows amplitude (*A*) versus frequency curves measured in water with the PT or magnetic excitation method. The measurements were performed for three different types of cantilevers (AC160, AC55 and USC-F5-k30) with different *f*_0_. Owing to the use of direct excitation methods, all the curves show a clean resonance peak without influence from any spurious resonances. These results demonstrate the capability of the developed magnetic excitation system to drive a cantilever with a megahertz-order *f*_0_.

The cantilever parameters such as spring constant (*k*), *f*_0_ and Q-factor (*Q*) before and after the bead attachment are summarized in Table 2. This table shows that the influence of the bead attachment becomes more evident as the cantilever size is reduced. For AC160, the influence is minor. However, for AC55 and USC, a 2–3 fold increase in *k* and 40–50% reduction in *f*_0_ are observed. In the meanwhile, the bead attachment increases *Q* due to the increase of the effective mass. This *Q* enhancement is particularly evident in the case of AC55. This is probably due to the difference in the cantilever shape: a nearly triangular shape for AC55 and a beam shape for AC160 and USC. Note that these changes in the parameters are dependent on the amount of the glue used for the bead fixation and hence show some variations.

**Table 2:** Influence of the magnetic bead attachment on the cantilever parameters and FM-AFM performance in water.

Parameters	AC160		AC55		USC-F5-k30	
	Before	After	Before	After	Before	After
*k* [N/m]	33	27.2	95.2	226	27.4	89.9
*f*_0_ [MHz]	0.147	0.125	1.76	1.01	3.88	1.93
*Q*	8.3	10.3	11.6	20.0	7.5	8.4
*F*_min_ [pN]	3.76	3.32	1.56	2.42	0.70	1.70
*A*_0_ [nm]	1.31	8.82	1.28	0.73	1.26	0.24

*k* was estimated from the thermal vibration spectra of the cantilevers. *f*_0_, *Q* and *A*_0_ were estimated by fitting the simple harmonic oscillator model to the curves shown in Fig. 6. *F*_min_ was calculated with these cantilever parameters and *B* = 100 Hz. As for the frequency range of the coil driver, we used range (1) for AC160 and range (3) for AC55 and USC.

With a small amplitude approximation, the minimum detectable force (*F*_min_) obtained by FM-AFM is given by^54^.

2 $${F}_{{\rm{\min }}}=\sqrt{\frac{4k{k}_{B}TB}{\pi {f}_{0}Q}},$$

where *k*_*B*_, *T* and *B* denote Boltzmann’s constant, an absolute temperature, and a measurement bandwidth, respectively. *F*_min_ estimated from the cantilever parameters are shown in Table 2. The result shows that the bead attachment slightly improves *F*_min_ for AC160 while it deteriorates *F*_min_ for AC55 and USC.

The maximum cantilever oscillation amplitude *A*_0_ estimated from the frequency sweep curves are also shown in Table 2. While *A*_0_ values obtained by the PT excitation are nearly the same for all the cantilevers, those obtained with the magnetic excitation show significant reduction with increasing *f*_0_. Thus, *A*_0_ obtained by the magnetic excitation is higher for AC160 but lower for USC than that obtained by the PT excitation. Such a reduction of *A*_0_ comes from the reduction of the output current of the coil driver and the size of the magnetic bead. For example, the coil drive current and the bead diameter were 0.5 A_rms_ and 20 μm for AC160 while they were 0.15 A_rms_ and 2 μm for USC. These differences lead to the difference in the excitation efficiency.

The typical *A* used for an atomic-resolution FM-AFM imaging in liquid is 0.2–0.5 nm while that for a 3D hydration measurement is 0.1–0.3 nm. Thus, the developed magnetic excitation system can be used for the 2D and 3D AFM measurements with an AC160 or AC55 cantilever. However, its excitation efficiency may not be sufficient for stable AFM measurements with a USC cantilever. In this study, we used a 2 *μ*m magnetic bead for a USC cantilever, and it is very difficult to increase this size due to the small dimension of the cantilever (length: 10 *μ*, width: 5 *μ*m). In the meanwhile, an increase of the output voltage results in an increase of temperature. Therefore, further improvement in the excitation efficiency should require improvements in the coil and heat radiation designs.

Based on these discussions, here we compare the performance obtained by the magnetic and PT excitation methods. For AC160, both methods provide a similar *F*_min_ but the magnetic excitation provides a much higher *A*_0_. Thus, the magnetic excitation is recommended especially for a sample having a relatively large surface roughness or an adhesive property. For AC55, the magnetic excitation provides a larger *F*_min_ than the PT method. Thus, the magnetic excitation method should be used mainly for a photo-sensitive sample. For USC, although we can drive the cantilever without inducing vibrations of spurious resonances, *A*_0_ may be too small to be used for a stable 2D imaging. At this stage, it may be used for a 1D or 3D force measurements with a relatively small *A*.

**Figure 7:**
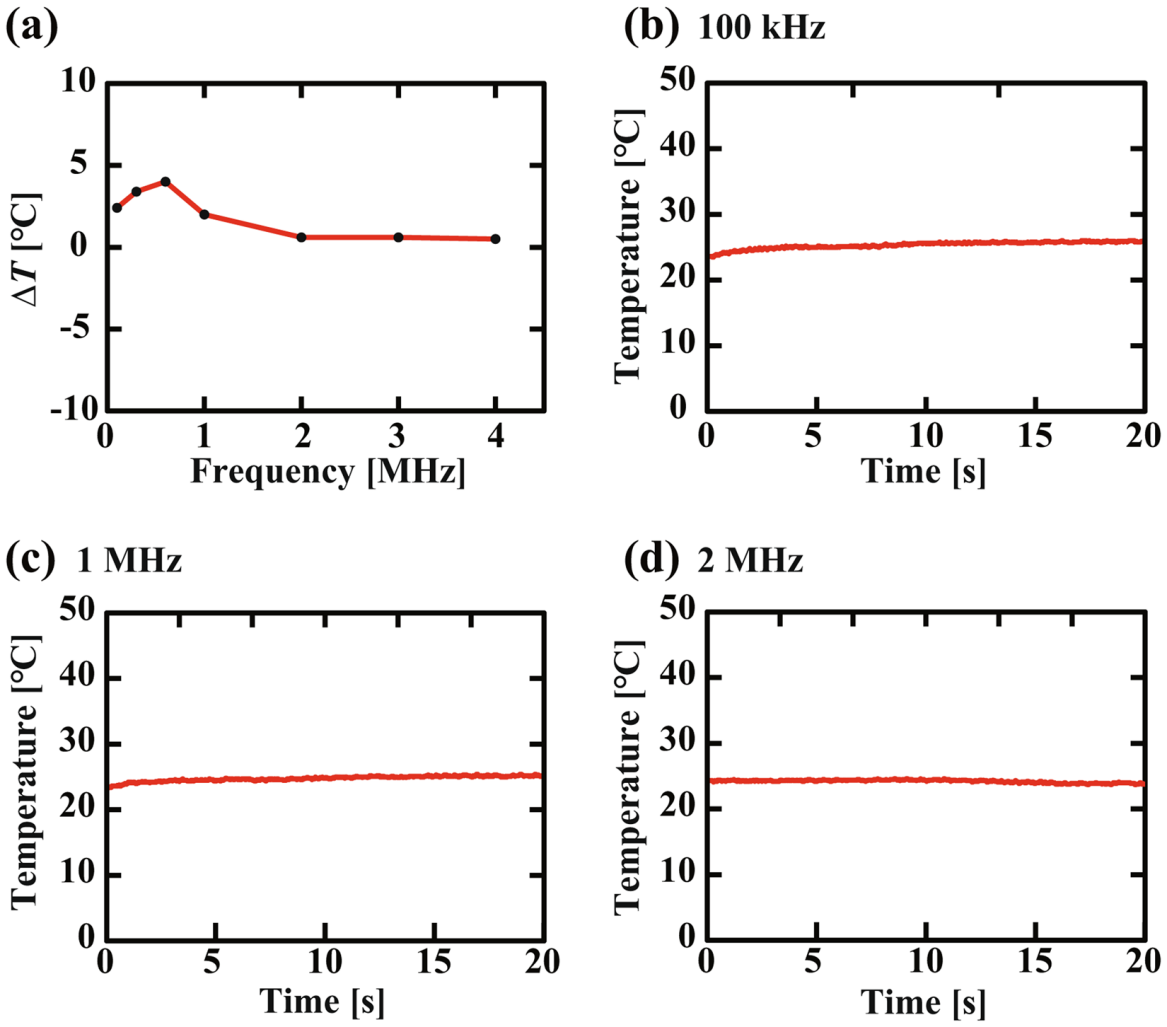
Temperature variations caused by the power dissipation at the coil. The temperature changes were measured in water on a mica substrate glued onto the sample holder using a thermocouple sensor. (**a**) Frequency dependence of the temperature change caused by an application of the magnetic field for 20 min. (**b–d**) Temperature variation during the first 20 min after the application of the magnetic field. Input voltage amplitude: 10 V (i.e., the maximum input). Power amp. gain: 20.

Figure 7(a) shows the frequency dependence of the temperature change caused by the heat dissipation at the coil during the application of the magnetic field for 20 min. The result shows that the temperature variation is kept less than 5 °C for all the frequency ranges. The temperature increase observed at the lower frequency ranges is due to the relatively large coil current as shown in Fig. 5(b). Such a temperature increase takes place only in the first a few minutes as shown in Fig. 7(b,c). For a frequency higher than 2 MHz, no temperature increase was detected as shown in Fig. 7(d). These results demonstrate that the influence of the heat dissipation on AFM imaging is negligible for many of the practical AFM applications.

**Figure 8:**
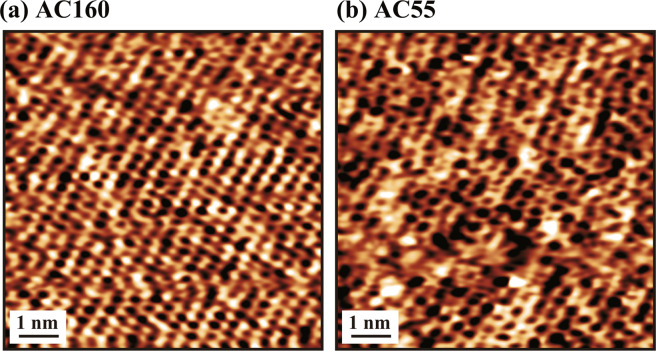
FM-AFM images of a cleaved mica surface obtained in PBS solution by the developed magnetic excitation system. The cantilevers used for the imaging include (**a**) AC160 and (**b**) AC55. For these cantilevers, we used the frequency ranges (1) and (3), respectively. Input voltage amplitude: 10 V (i.e., the maximum input). Power amp. gain: 20.

Figure 8 shows FM-AFM images of a cleaved mica surface obtained in phosphate buffered saline (PBS) solution by the developed magnetic excitation system. The imaging was performed with different cantilevers including AC160 and AC55. For both cases, the obtained images show atomic-scale contrasts with a ~0.5 nm periodicity. Atomic-resolution FM-AFM imaging in liquid requires various factors such as low-noise and highly stable cantilever deflection detection, cantilever oscillation control and tip-sample distance regulation. Among them, stable excitation of a cantilever is one of the essential conditions for an atomic-resolution FM-AFM imaging. Therefore, the atomic-resolution images shown in Fig. 8 demonstrate that the noise performance and stability of the developed magnetic excitation system are sufficient for an atomic-resolution imaging in liquid.

## Conclusions

In this study, we have developed the wideband magnetic excitation system for driving a small cantilever with a megahertz-order *f*_0_ in liquid. In the system, an excitation coil was implemented in a sample holder and a magnetic bead was attached on the front side of a cantilever. On the bead, an EBD carbon tip was fabricated for an AFM imaging in liquid. In the coil driver, differentiation circuits were used for compensating the frequency dependence of the coil impedance and thereby keeping the output current constant within a specified frequency range. By implementing several differentiation circuits with different frequency ranges, we have enabled to drive various cantilevers with different *f*_0_ up to 4 MHz with sufficient excitation efficiency. In contrast to the conventional coil driver consisting of a closed-loop circuit (i.e., *VI* converter), the developed coil driver consists of an open-loop circuit. Thus, it can be stably operated regardless of the electrical properties of the excitation coil. This capability should be useful for the future improvements in the coil design to enhance excitation efficiency or to suppress the heat dissipation. The developed magnetic excitation system was combined with an optical beam deflection sensor using a 830 nm super luminescent diode. Thus, the developed system allows to perform AFM measurements without irradiating any visible light to the sample surface. The applicability of the developed system to an AFM experiment was demonstrated by measuring the cantilever resonance curves and atomic-resolution imaging of a mica surface in PBS solution with various cantilevers including those with a megahertz-order *f*_0_.

## Methods

The imaging and fabrications of the cantilevers (Fig. 1) were performed with a FIB-SEM (Helios CX, Thermo Fisher Scientific). The magnetic bead (MQP-S-11-9-20001-070, Neo Magnequench) was attached to the cantilever with a micromanipulator (Axis-Pro, Microsupport). The frequency dependence of the output voltage and current of the coil driver (Fig. 5) was measured by a frequency response analyzer (FRA5097, NF). At the output stage of the developed coil driver, we used a commercially available power amp. (HSA4101, NF). A home-built AFM system with a low noise cantilever deflection sensor^55–57^ and a high-stability photothermal cantilever excitation system^15,57^ was used for the AFM measurements. To control this AFM system, we used a commercially available AFM controller (Nanonis OC4 and RC4, SPECS). The atomic-resolution AFM imaging (Fig. 8) was performed in the FM detection mode with a constant cantilever oscillation amplitude. The sample was prepared by cleaving a round disk of muscovite mica (01877MB, SPI Supplies). The PBS solution was prepared by dissolving a tablet of BPS (Fluka) into 200 ml of Milli-Q water (Elix 3/ Milli-Q Element system, Millipore).

## Supplementary Information

Supplementary Information.
