# Supplementary material for: Wideband Magnetic Excitation System for Atomic Force Microscopy Cantilevers with Megahertz-Order Resonance Frequency
Source: Sci Rep. 2020 Jun 4;10:9133. doi: 10.1038/s41598-020-65980-4 (PMC7272457; doi:10.1038/s41598-020-65980-4)
Supplement: Supplementary file 1 — Supplementary Information. [file 41598_2020_65980_MOESM1_ESM.pdf]

Supplementary Information

Wideband Magnetic Excitation System for Atomic Force Microscopy Cantilevers with Megahertz-Order Resonance Frequency

Kaito Hirata[1], Takumi Igarashi[1], Keita Suzuki[1], Keisuke Miyazawa[1,2], and Takeshi Fukuma[1,2,*]

[1]Division of Electrical Engineering and Computer Science, Kanazawa University, Kakuma-machi, Kanazawa, 920-1192, Japan

[2]Nano Life Science Institute (WPI-NanoLSI), Kanazawa University, Kakuma-machi, Kanazawa 920-1192, Japan

*fukuma@staff.kanazawa-u.ac.jp

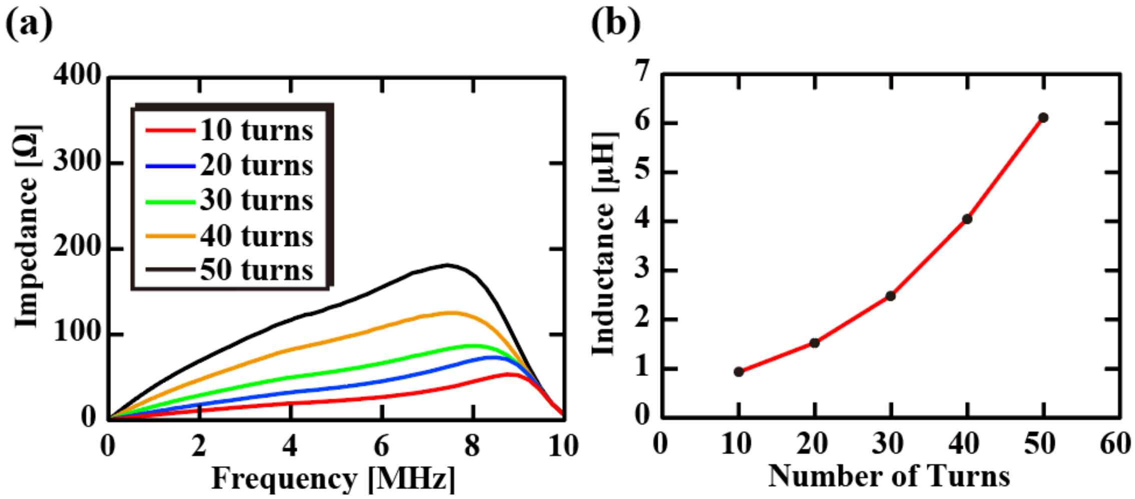

Figure S1: Dependence of the coil characteristics on the number of turns. (a) Coil inductance. (b) Coil impedance estimated from the linear slope at the low frequency range (< 1 MHz) of the curves shown in (a).

Figure S1a shows the coil impedance versus frequency curves and their dependence on the number of turns (N). Ideally, the coil impedance is given by ωL. Thus, it linearly increases with frequency. This is true at a relatively low frequency range (< 1 MHz). Above this frequency range, the slope gradually decreases with increasing frequency. This dependence is compensated by the non-linear gain response of the differentiation amplifier shown in Figure 4e.

From the slope of the linear part, we can estimate the inductance of the coil as shown in Figure S1b. This result shows that the inductance increases with increasing N. Theoretically, the inductance of an

infinitely long solenoid coil increases in proportion to N^2. However, due to the small number of turns and small aspect ratio of the coil shape, it does not follow this dependence. In this study, we used several coils with $N = 50$ turns. However, as the coil was handmade, the inductance shows some variations from 5 to 10 μH.
